# Possible Recycling of Cigarette Butts as Fiber Modifier in Bitumen for Asphalt Concrete

**DOI:** 10.3390/ma13030734

**Published:** 2020-02-06

**Authors:** Md Tareq Rahman, Abbas Mohajerani, Filippo Giustozzi

**Affiliations:** School of Engineering, RMIT University, Melbourne 3000, Australia; abbas.mohajerani@rmit.edu.au (A.M.); filippo.giustozzi@rmit.edu.au (F.G.)

**Keywords:** recycling, cigarette butts, bitumen, asphalt concrete, environmental sustainability, waste management

## Abstract

Littering waste is among the top environmental issues in the world, and the management of the waste has turned into a challenge in almost every city. It has been reported that 75% of smokers dispose of their cigarette butts (CBs) on the ground, even in public places. Researchers have discovered that CBs make up more than one-third of the total littered waste on the planet. Cigarette butts predominantly consist of a cellulose acetate fiber (plastic)-based filter wrapped in paper. Waste CBs contain burnt tobacco and tar, along with many other toxic chemicals. They take years to biodegrade depending on the environmental conditions, and toxic chemicals leach out and contaminate the environment. As part of an ongoing project, this paper presents a novel and sustainable technique to recycle cigarette butts in bitumen for the construction of flexible pavements. In this research, CBs have been pre-processed and mixed with bitumen classes C320, C170, and PMB A10E as a fiber modifier. Comprehensive laboratory investigations, including a penetration test, softening point test, and viscosity test, have been performed along with a binder drain off test to evaluate the performance of the modified samples. During this investigation, samples were prepared with 0.3% cellulose fiber, 0.2%, 0.3% 0.4%, and 0.5% CBs. The results of the CB-modified samples were compared with the sample with cellulose fiber and fresh bitumen (0% fiber). The results show that the physical and rheological properties of bitumen incorporating CBs improve significantly, and CBs could be used instead of virgin cellulose fiber as a fiber modifier.

## 1. Introduction

### 1.1. Cigarette Butt Pollution

**Figure 1:**
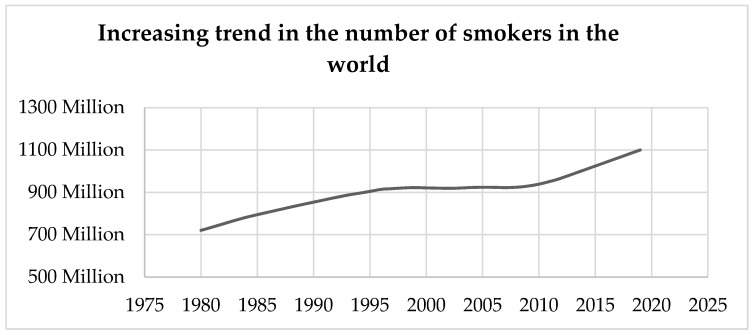
Increased rate of the number of daily smokers in the world from 1980–2019; adapted from the Institute for Health Metrics and Evaluation (2014) and World Health Organization [3,4].

**Figure 2:**
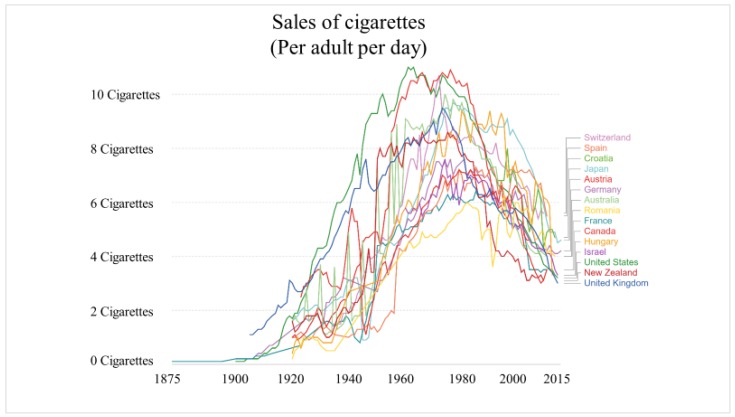
Sales of cigarettes in some of the countries of the world [1,2,4].

Cigarette Butts (CBs) are the lower part of the cigarette, which generally comprises the filter. Max Roser and Hannah Ritchie investigated the number of smokers in the world and concluded by mentioning that there had been a drastic increase in the number of smokers around the world. The number had already crossed 1 billion by 2012 [1,2]. In 2018, the World Health Organization summarized the number of smokers in the world as totaling 1.1 billion, even after numerous health awareness campaigns [3]. Notwithstanding the statistics, the actual number of smokers is probably much higher and continuing to increase, as shown in Figure 1, which was adapted from the Institute for Health Metrics and Evaluation (IHME) 2014 and the World Health Organization [3,4]. The production of cigarettes has increased to support the increasing need. A recent study shows that the current production of cigarettes is 6 trillion, of which 5.8 trillion are consumed every year [5]. According to the World Health Organization, production will reach 9 trillion by 2025 [5,6,7]. Figure 2 shows the sale of cigarettes per adult smoker per day in some of the countries of the world [1,2].

However, these smoked cigarette butts have a damaging impact on the environment. “The Terramar project” has determined that throughout the world, 2.3 million cigarette butts are littered every minute, and out of ten discarded CBs, one CB is going into a water body [8]. The research by “truth initiative” (2017) has found that cigarette butts make up 38% of the total littered waste [9]. It has been reported that around 75% of smokers throw their CBs on the ground, even in public places.

**Figure 3:**
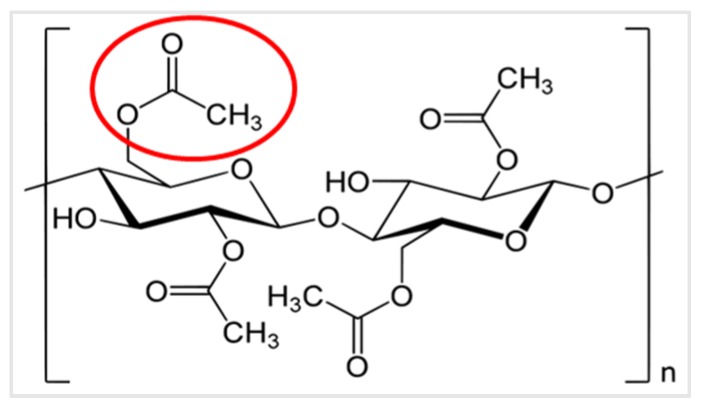
Polymer structure of cellulose acetate. One of the cellulose acetate groups is marked with a red circle.

The littered part of a cigarette mostly contains a filter, which is predominantly based on cellulose acetate fiber wrapped with paper and termed the cigarette butt. The chemical structure of cellulose acetate is shown in Figure 3.

Burnt CBs contain nicotine, tar, CO, arsenic, and many other toxic chemicals [10,11]. When CBs are disposed of on the ground, chemicals start leaching into the environment [12,13,14]. These chemicals contaminate the soil, plants, animals, and water bodies [12]. When CBs are in contact with flowing water, the chemicals spread faster and cause contamination to life in the water and deteriorate the water quality.

The management of waste CBs is a pressing issue. Most countries manage cigarette butts by disposing of them in landfills along with other waste, while, in others, they are incinerated, which generates toxic fumes and leads to severe air pollution [15,16]. Research has been carried out to recycle CBs in different manufactured materials. A recent approach to recycle CBs along with other waste products like bagasse fiber, palm oil fruit bunch fiber in noise control materials and sound absorption property has found promising results [17,18]. Teixeira et al. (2016) investigated a method to recycle CBs in cellulose pulp for the paper industry; however, the management of effluent and organic materials was found to be challenging [19].

### 1.2. Use of Waste Cigarette Butts in Construction Materials

The recycling of CBs and turning this waste into a resource can be a solution to cigarette butt pollution. Researchers have developed methods to recycle different waste materials as aggregates in construction materials [20]. Góra et al. (2019) utilized recycled LCD (liquid crystal display) as fine aggregate in concrete. Samples exhibited good freeze-thaw resistance and performed similarly to C50/60 concrete [21]. Based on a comprehensive study, Mohajerani et al. (2016) proposed that the CB pollution problem can be solved globally by incorporating 1% CBs in 2.5% of the world’s brick production. The study involved incorporating various percentages of CBs in clay bricks, including 2.5%, 5%, 7.5%, and 10% [14,22,23,24]. Furthermore, Mohajerani et al. (2017) revealed a new method to recycle CBs in asphalt concrete for the construction of flexible pavement [10]. In their study, cigarette butts were encapsulated by paraffin wax and bitumen and the encapsulated CBs were incorporated in dense asphalt. This comprehensive study investigated the effects of incorporating encapsulated CBs as aggregate in different percentages of up to 15 kg/m^3^, on the physical and mechanical properties of dense asphalt and found very promising results [10]. This paper is part of a study from the same ongoing research at RMIT University focusing on exploring a method to recycle CBs in bitumen as a fiber modifier that will introduce a unique form of recycling approach. This research aims to recycle CBs as a fiber modifier for bitumen for the construction of flexible pavements. The preparation and incorporation of CBs in different classes of bitumen are investigated, and some results from the ongoing exploration are presented and discussed in this paper.

### 1.3. Use of Waste Materials in Bitumen

**Figure 4:**
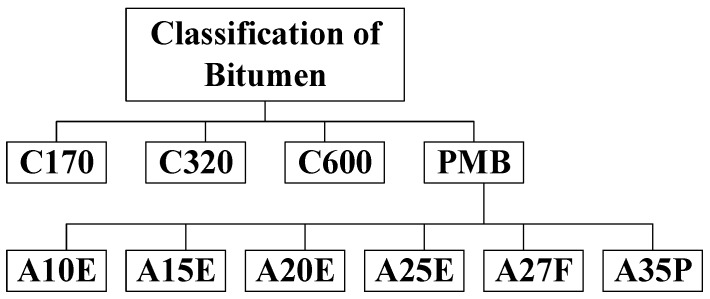
Classification of bitumen according to Australian standard for the construction of pavements.

Bitumen is a viscoelastic complex hydrocarbon that is black or brown. Although there are a few natural sources of bitumen available, bitumen is generally sourced from crude oil refineries [25]. Because of its waterproofing and viscoelastic nature, bitumen is used as the binder for the construction of flexible pavement all over the world. Bitumen can be classified into three categories: penetration grade, performance grade, and viscosity. Nowadays, bitumen classification based on the viscosity grade is gaining popularity, and the available types according to the Australian Standard which is named after the typical viscosity of bitumen at 60 °C except for PMB class (polymer modified bitumen) for the construction of flexible pavements are provided in Figure 4 [26].

Around the world, researchers are working to improve the properties of these materials to ensure sustainability in the pavement construction sector [25,27,28]. Recycling waste materials in asphalt is recognized as a very efficient method, as it improves the pavement quality, and, at the same time, helps to manage and recycle different waste products [27]. Many researchers have investigated the use of different waste materials in bitumen. Plastic and polymer-based modifiers have been used extensively for a long time. Many industries have adopted plastic rubber and polymer modified bitumen for the construction of roads [28,29,30], while numerous researchers have investigated the use of regular household residue like waste cooking oil in bitumen. In some cases, they have recommended an optimum amount of waste cooking oil in bitumen, which is up to 5% (by weight), to ensure that any resultant compromise in the performance is minimized [28,31]. Intending to achieve better aging resistance, researchers have used palm oil fuel ash (POFA) to modify bitumen and found that POFA in bitumen can work as a rejuvenator for the binder [28,32,33]. Different types of fiber have been used in construction materials to alleviate the global waste management issue [34]. A number of studies have found that fiber can improve the performance of bitumen [34,35,36,37]. Researchers have investigated the use of synthetic fibers like polymer fiber, steel fiber, and carbon fibers in asphalt concrete [34]. It has been found that carbon fiber can improve the electrical property of asphalt but compromise the mechanical performance of asphalt concrete while steel fiber improves the stability of asphalt [38,39]. Industry uses cellulose fiber to reduce binder drain off during the transportation of the mix from the plant to the construction site [40,41]. As cigarette butt filter is cellulose acetate based fiber, it can be a potential replacement of natural cellulose fiber used in stone mastic asphalt. Recycling suitable waste in bitumen in a proper manner is a sustainable way to contribute to solving the waste management problem of the world.

## 2. Materials and Method

The types of bitumen were selected based on the most popular types of bitumen used in asphalt concrete in Australia. Samples were prepared in laboratory conditions with CBs, and the results were compared with the control sample and the samples prepared with conventional cellulose fiber. Incorporation of cellulose fiber in stone mastic asphalt is a common practice in the industry to prevent bitumen drain off during mixing and the transportation of materials.

### 2.1. Materials

In this research, three types of bitumen were used to investigate this novel approach. Bitumen class 320, 170, and PMB A10E were used as a binder. Nowadays, the industry uses cellulose fiber with bitumen for the construction of stone mastic asphalt (SMA). Fiber prevents the drain-off of the bitumen during transportation to the site. Cellulose fiber and shredded cigarette butts were used to modify bitumen in this research. Cigarette butts were pre-processed before blending with bitumen. The industry standard for the binders is provided in Table 1.

#### Pre-Processing of CB

**Figure 5:**
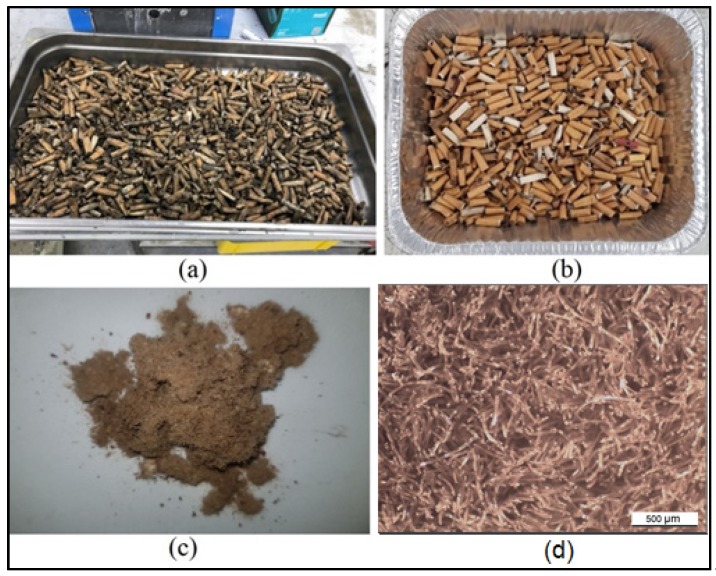
Cigarette butts (**a**) before and (**b**) after oven drying and the removal of excess tobacco and dirt; (**c**) ground cigarette butts (CBs) used for sample preparation; (**d**) view of CB fibers under optical microscope.

The cigarette butts (CBs) used in this study were supplied by Butt out Australia Pty Ltd. After collection, the CBs were stored in a plastic tub. The cigarette butts were dried in the oven for 24 h, and excess tobacco was removed from the CB. The dried CBs were shredded into ground fiber with a grinder in sealed and controlled conditions. The ground CBs were later blended with bitumen samples. Figure 5 shows a part of the pre-processing of the CBs.

### 2.2. Method

#### 2.2.1. Blending of Bitumen with Fibers and Sample Preparation

The fibers were blended with three different types of bitumen. The blending process was replicated three times to confirm repeatability. A total of eighteen samples were prepared for each blending approach. From each type of bitumen, six types of samples were prepared. The first sample was the control sample without any fiber blended with the sample. The second sample comprised 0.3% cellulose fiber. The rest of the samples contained 0.2%, 0.3% 0.4%, and 0.5% shredded CBs, respectively. Table 2, Table 3 and Table 4 show the blending design for Bitumen Class A10E, C320, and C170 with cellulose fiber and CB fiber for blending approach 1, approach 2 and approach 3 consecutively.

**Figure 6:**
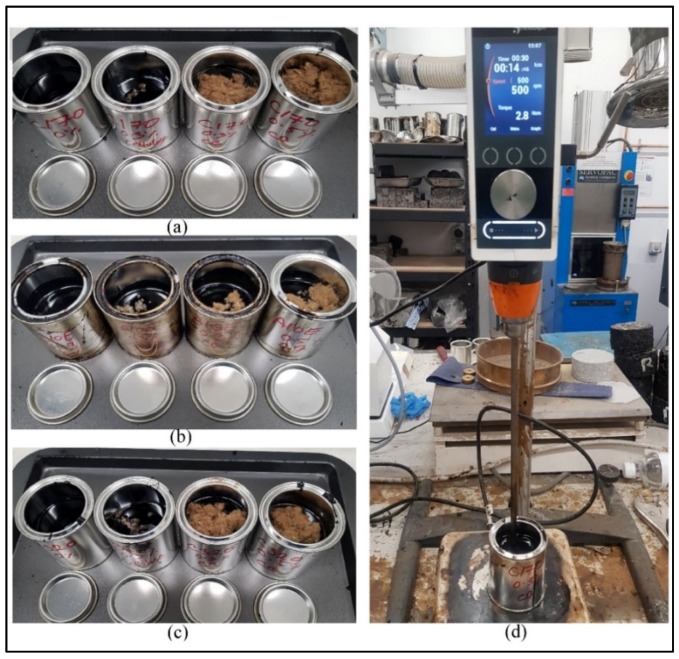
(**a**) Bitumen C170, (**b**) Bitumen A10E, (**c**) Bitumen C320, and (**d**) blending of bitumen with fibers using the shear mixture.

The samples were blended with a shear mixture in laboratory conditions. The blending temperature was set at 160 °C, and the rotation speed was 500 rpm. After blending all the samples, they were labeled and prepared for laboratory investigations. The shear mixture was used to blend bitumen with cellulose and CB fiber. Nowadays, the industry uses cellulose fiber with bitumen for the construction of stone mastic asphalt (SMA). Fiber prevents the drain-off of the bitumen during transportation to the site. Figure 6 exhibits few of the samples before the mixture and the blending process.

#### 2.2.2. Laboratory Tests

**Figure 7:**
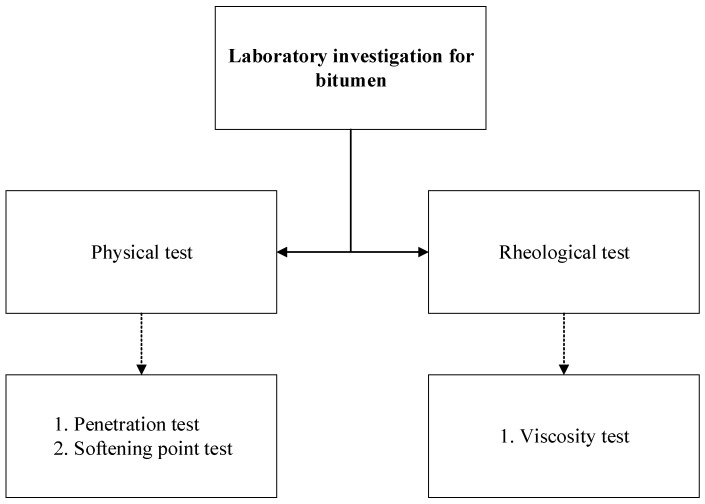
Adapted laboratory test flow for bitumen followed in this research.

All the samples were assessed in the laboratory following the AASHTO, ASTM, and Australian Standards (AS). The laboratory tests included a penetration test, softening point test, and viscosity test. The average result of at least three replicate tests was used for the comparison. The details of the laboratory investigation plan are shown in Figure 7. In addition, a binder drain-off test was performed on the asphalt samples prepared with fiber modified bitumen to assess the drain down properties.

#### 2.2.3. Penetration Test

**Figure 8:**
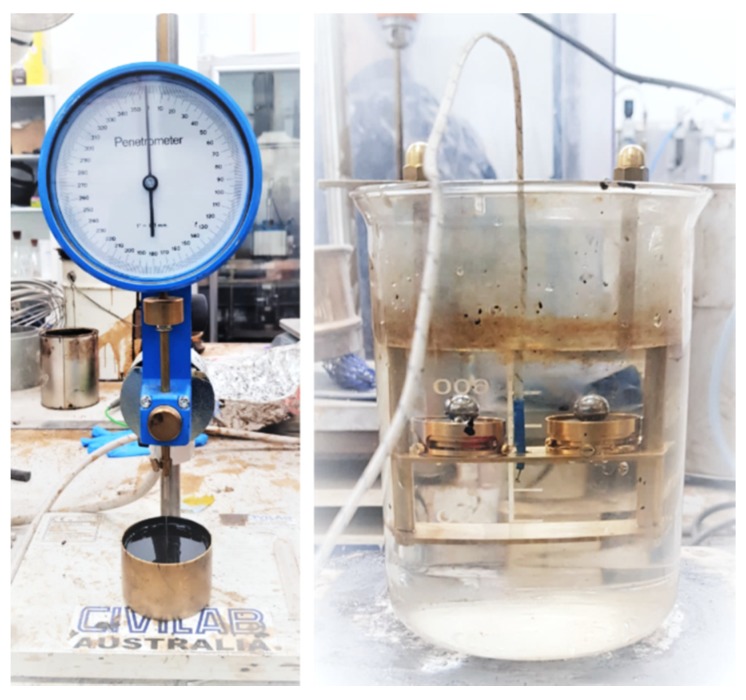
Penetration test (on left) and softening point test (on right) of a sample carried out in this study.

The penetration test was carried out according to AASHTO T 49 and AS 2341.12. During the test, samples were collected in penetration cups and conditioned in the water bath at 25 °C temperature for 2 h. Later, a penetrometer was used to determine the penetration value of the samples. The results were compared with the control sample without any fiber. Figure 8 shows the penetration test of a sample in the laboratory.

#### 2.2.4. Softening Point Test

The softening point test of all twelve samples was carried out with a ring and ball apparatus. The lab test was conducted according to AASHTO T 53 ASTM D 36 and AG: PT/T131. Figure 8 shows the softening point test carried out for this research.

#### 2.2.5. Viscosity Test

**Figure 9:**
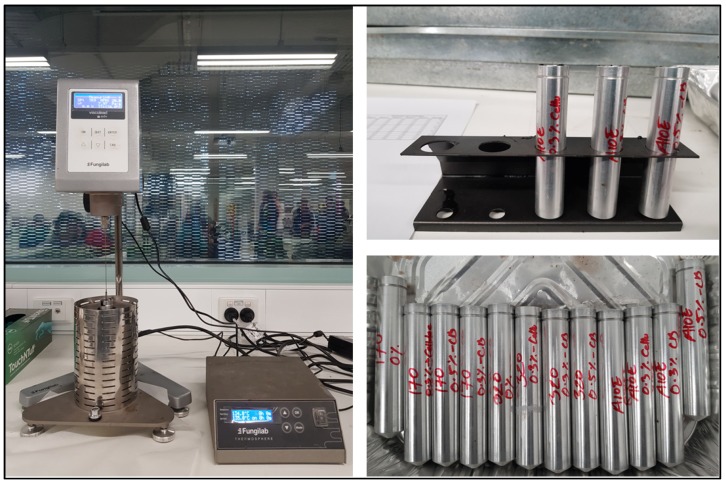
Viscosity test of samples used in this study. Use of rotational viscometer to assess viscosity (left); cylindrical containers used to store and perform viscosity test of bitumen (right).

The viscosity test was conducted on all the samples using a Fungilab (Barcelona, Spain) rotational viscometer according to AASHTO T 316, ASTM D 4402, and AG: PT/T111. Figure 9 shows the viscosity test of the binder carried out in the laboratory.

## 3. Results and Discussion

The results of the laboratory tests were observed and analyzed; the outcome of which was promising. The outcome of the analysis is presented and discussed in this section.

### 3.1. Penetration and Softening Point Test Results

The average result of the penetration and softening point tests for all blending approaches are mentioned in Table 5.

In the results, it can be observed that, on average, in the case of bitumen PMB A10E, the control sample with no fiber present had an average penetration value of 62.2 dmm (deci-millimeter). When 0.3% of cellulose fibers was added, an increase in the penetration value was observed. However, after the addition of shredded CBs as fiber in the bitumen A10E, a slight decrease in the penetration value was found. As more CBs were added, the penetration values started to decrease and maintained the range of 61–62 dmm. In the case of bitumen C320, with the addition of CBs fiber, the penetration value increased, and penetration value stayed within 40–43 dmm. Bitumen C170, without any fibers, possessed the maximum penetration value of 63.3 dmm. On the other hand, when Bitumen C170 was modified with 0.2% CBs fibers in the mix it exhibited the highest penetration value among all the modified C170 bitumen samples, and the increase in the presence of CBs in the mix reduced the penetration value. The probable reason is the soft nature of C170, and the absence of polymer content compared to the PMB class. The results suggested that the use of CBs fibers in bitumen is not significantly detrimental to the penetration property. In the results, it can be observed that, on average, in the case of bitumen PMB A10E, the control sample with no fiber present has a penetration value of 62.8 dm. When 0.3% of cellulose fibers was added, an increase in the penetration value was observed. However, after the addition of shredded CBs as fiber in the bitumen A10E, a slight decrease in the penetration value was found. As more CBs were added, the penetration value started to decrease. In the case of bitumen C320, with the addition of CB fiber, the penetration value increased. Bitumen C170 without any fibers, possessed the maximum penetration value of 66 dm. On the other hand, when Bitumen C170 was modified with 0.3% fibers in the mix, it exhibited the highest penetration value among all the modified C170 bitumen, and the increase in the presence of CBs in the mix reduced the penetration value. The results suggested that the use of CBs fibers in bitumen is not significantly detrimental to the penetration performance and a 0.3% addition of CBs does not compromise the typical penetration range of bitumen. Variance of the penetration test result was calculated to understand the mean differences of the result in all three approaches. Results from bitumen PMB A10E shows variance of 0.2, C320 having variance 1.42 and C170 having variance of 0.75. This shows that despite of modification of bitumen with CBs, results are very close to each other and CBs fiber do not damage the penetration property of bitumen.

After analyzing the softening point of samples, it has been found that, use of CBs as fiber in PMB A10E decreases the softening point slightly and maintains a range between 89.5–90 °C. Variance for softening point difference in three blending was found 0.25. Bitumen C320 modified with CB has softening point around 45 °C and variance was as low as 0.03. Softening point of bitumen C170 modified with CB stayed very close to 41 °C and variance among three approaches was 0.11. This shows that results are very similar to the control sample and all these samples might be suitable for the construction of flexible pavement.

### 3.2. Viscosity Test

The viscosity test was carried out at two different temperatures, 135 °C and 165 °C. The results are given in Table 6.

The results show a positive resemblance in the viscosity of the samples. In the case of PMB A10E, the control sample without any fibers, the viscosity was 6.49 Pa. s at 135 °C. However, when PMB A10E was modified with cellulose fibers, a slight increase in viscosity was noticed. When it was modified with shredded CBs, an increase in the viscosity was found with the increase in the quantity of CBs in the mixture. The presence of the polymer content in the bitumen has a vital role in terms of higher viscosity.

**Figure 10:**
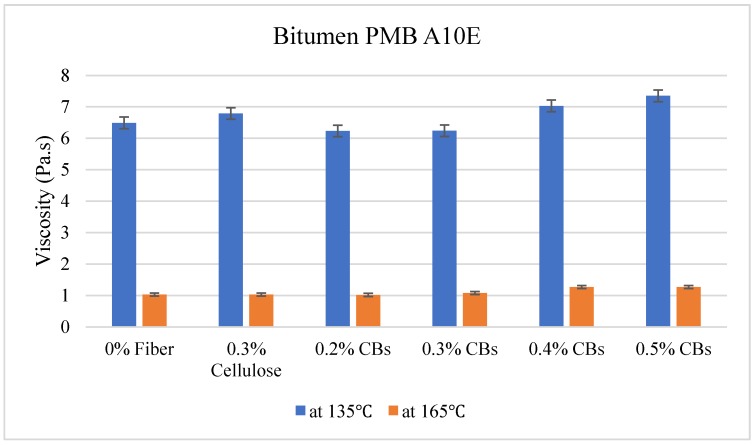
Comparative viscosity of Bitumen PMB A10E modified with different types of fiber.

**Figure 11:**
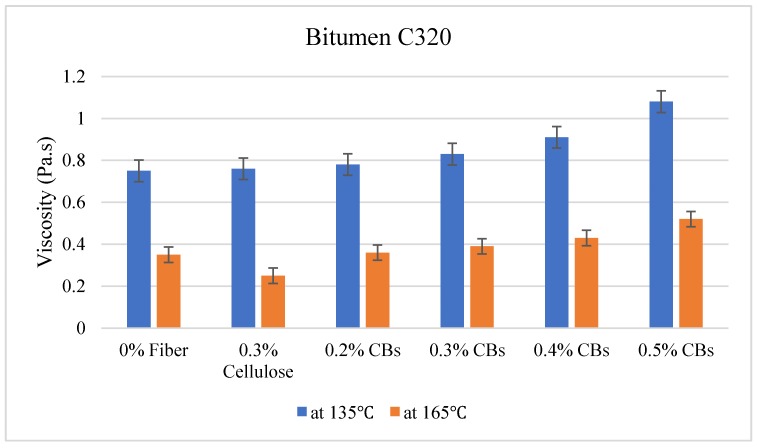
Comparative viscosity of Bitumen C320 modified with different types of fiber.

**Figure 12:**
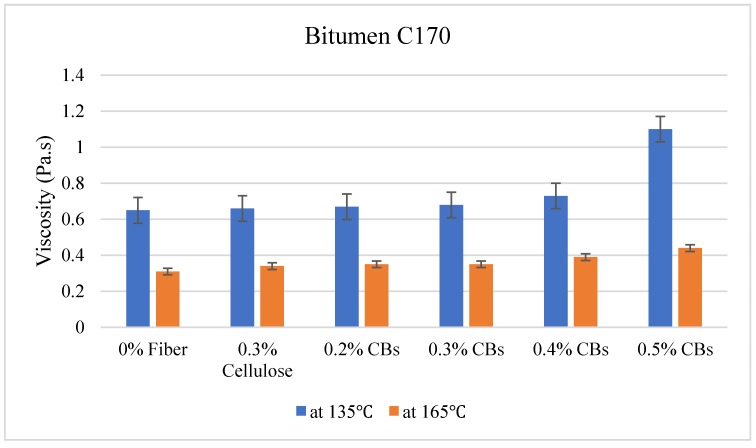
Comparative viscosity of Bitumen C170 modified with different types of fiber.

On the other hand, Bitumen C320 and C170, which have low viscosity compared to PMB A10E because of the absence of polymer content, showed an increase in the viscosity with the increase of CBs in the mixture. Figure 10, Figure 11 and Figure 12 illustrate the comparative average viscosity from different blending approach for different types of bitumen.

**Figure 13:**
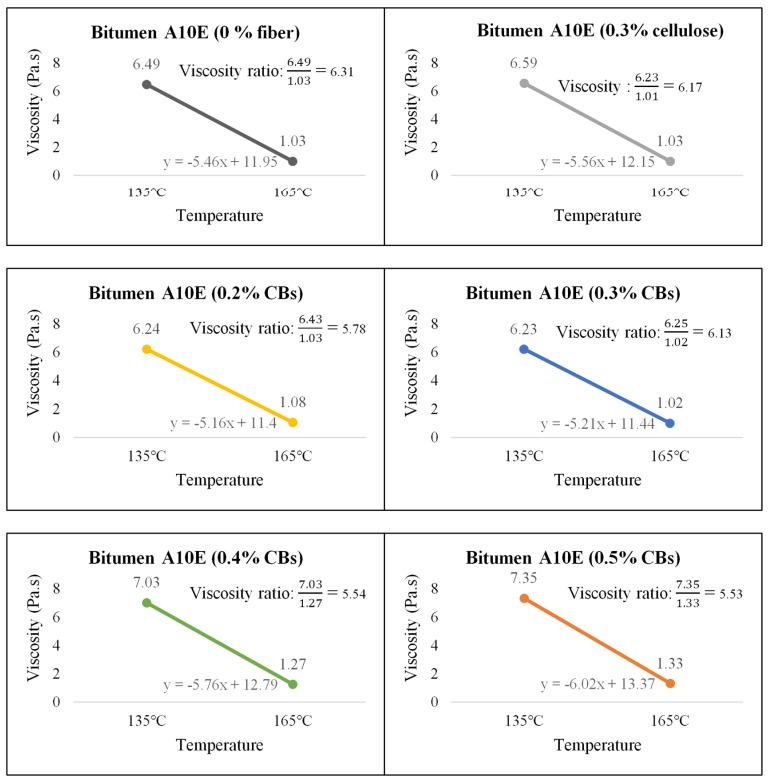
Viscosity ratio and slope of Bitumen PMB A10E with cellulose fiber and different % of CB content.

**Figure 14:**
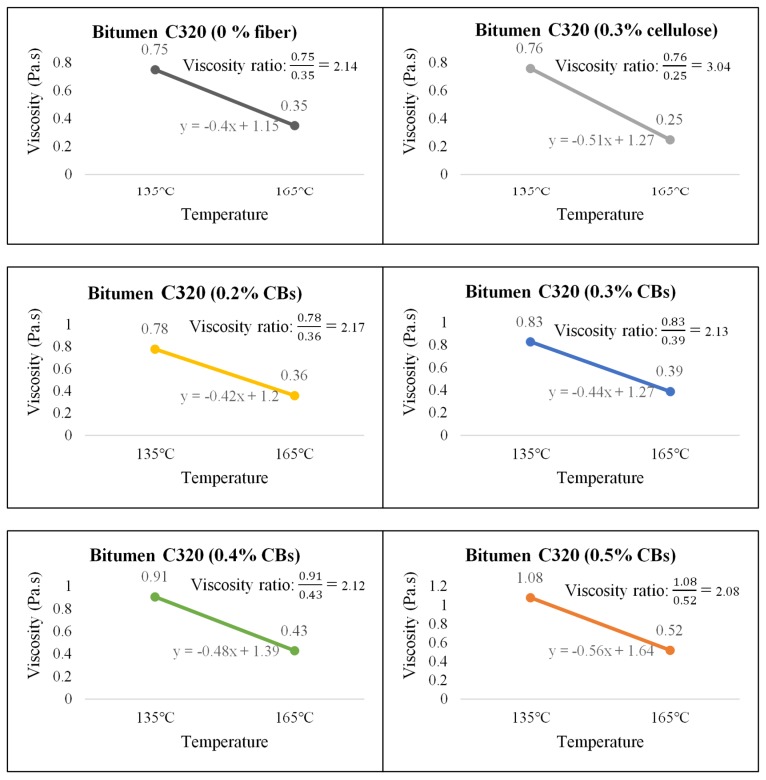
Viscosity ratio and slope of Bitumen C320 with cellulose fiber and different % of CBs content.

**Figure 15:**
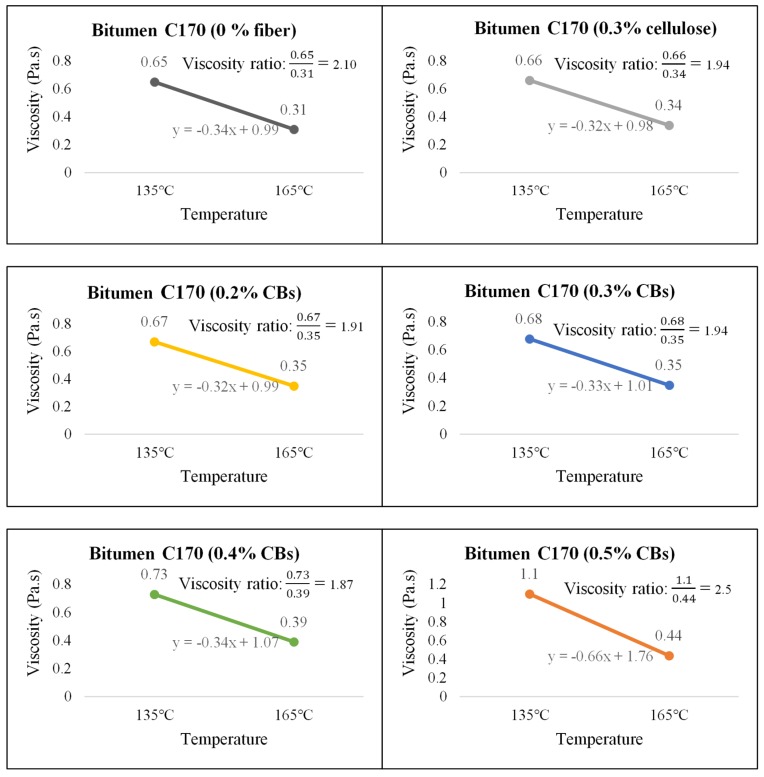
Viscosity ratio and slope of Bitumen C170 with cellulose fiber and different % of CBs content.

The viscosity ratio, as a relative change in the state of flow and linear slope for two different temperatures (135 °C and 165 °C), is provided to understand the slope condition of the temperature susceptibility graph. A higher value for the slope indicates a steep slope; hence, the sample is comparatively more vulnerable to temperature change. A lower value for the slope indicates a comparatively flat slope, and the sample is less susceptible to temperature change. Figure 13, Figure 14 and Figure 15 show the viscosity ratio and the slope of the samples due to the change of viscosity from 135 °C to 165 °C.

The viscosity ratio of the samples prepared with bitumen PMB A10E shows that the addition of 0.3% CBs in the binder has the highest viscosity ratio of 6.13 among other modified bitumen samples. In terms of temperature susceptibility, 0.3% CBs in bitumen keeps the binder very similar to the control sample. Considering the slope condition, the sample with 0.3% CBs has the slope closest to the control sample as well.

In case of bitumen C320, it has been found that the sample with 0.3% and 0.4% CB shows a very similar temperature susceptibility trend compared to the control sample. Considering the slope condition, 0.2% and 0.3% CBs-modified samples have a slope very close to the control sample.

The viscosity ratio of the samples prepared with bitumen C170 shows that the addition of 0.4% CBs in the binder has the lowest viscosity ratio of 1.87. Even the sample with 0.5% CBs had a viscosity ratio of 2.5, which is very close to the viscosity ratio of the control sample which is 2.10. Nevertheless, considering the linear slope condition, the sample modified with 0.2% and 0.3% CBs showed the closest slope after the control sample.

### 3.3. Binder Drain off Test

The industry maintains 6–7% of bitumen in the SMA during the construction of flexible pavements (VicRoads 2012). Because of the higher percentage of bitumen in SMA, binder drain off is significant in this type of asphalt. The loss of binder occurs during the mixing process and transportation to the site. Cellulose fiber is used in SMA to reduce the loss of bitumen because of binder drain off (VicRoads 2012). Three types of samples were prepared for this investigation: samples without any fiber, samples with cellulose fiber, and samples prepared with CBs modified bitumen. The samples were prepared and mixed at 185 °C following VicRoads (2012) specified gradation and materials as mentioned in Table 7.

**Figure 16:**
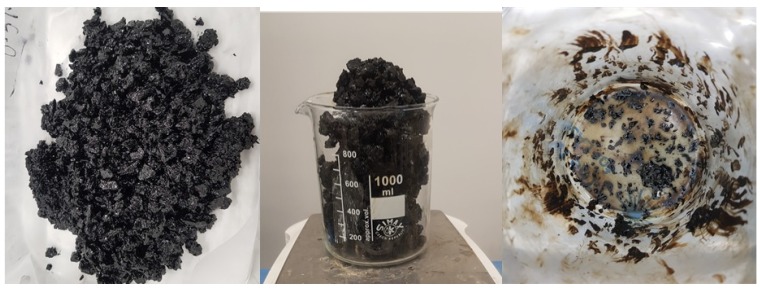
Binder drain off process. Asphalt mix prepared with CBs modified bitumen (**left**); preparation of binder drain off test (**middle**); drained off binder with fine aggregates retained at the bottom of glass flask (**right**).

The binder drain off test was performed on stone mastic asphalt (SMA) samples according to Austroads test standard AG: PT/T235. Part of the binder drain off process is shown in Figure 16.

**Figure 17:**
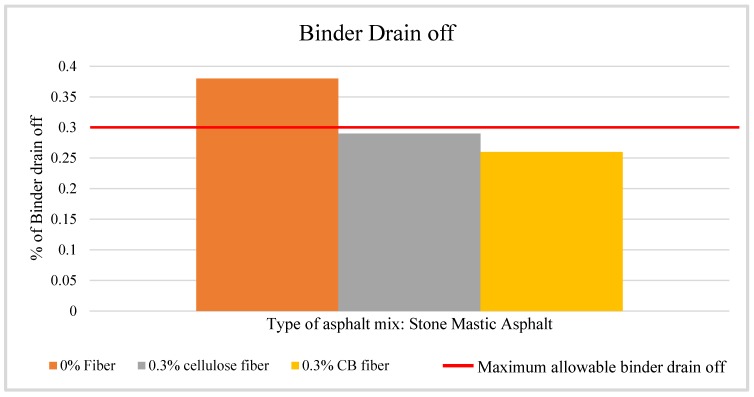
Relative view of ‘Binder drain off’ of asphalt mixture prepared with bitumen modified with different types of fiber.

Table 8 shows the results of the binder drain off tests. The bitumen sample prepared with 0.3% CBs fiber shows 0.26% of binder drain off, while the sample prepared with conventional cellulose fiber has 0.29% of binder drain off. These results show that the CBs modified binder has a lower drain off; hence, the loss of binder during transportation will be less. The industry maintains a maximum binder drain off of 0.3% [26,40]. Figure 17 shows the relative difference in the percentage of binder loss with the maximum recommended limit practiced by industry, which helps one to understand the significance of the use of CBs in bitumen as fiber in terms of improving the binder drain off property.

## 4. Conclusions

This study has investigated the possible recycling of cigarette butts in bitumen as a fiber modifier for the construction of flexible pavements. The materials were selected considering industry standards and all the tests were carried out according to the Australian Standards, ASTM, and Austroads guidelines.

The penetration results indicated that, although there is a slight decrease in the penetration, the results are very close to the standard range for the respective bitumen. The typical penetration value for Bitumen C320 is a minimum of 40 dmm, and all the samples modified with CBs met this condition. On the other hand, the typical penetration value for Bitumen 170 is 62 dmm. However, the penetration result of bitumen C170 was found to be slightly lower than 62 dmm. In the case of PMB A10E, the penetration values are very close to the control sample and higher than 62 dmm. It can be concluded from the penetration test that Bitumen C320 and PMB A10E is the most appropriate type of bitumen to be modified with CBs without compromising the penetration property.

The softening point of the samples slightly changed after the modification with fiber. However, even after the decrease, it was found that the results are similar to the control sample and within the typical range. During the comparison, it was observed that with the increase in the CBs in the mixture, the softening point moderately decreases except for the case of bitumen C320. The samples with 0.2 and 0.3% CBs have a softening point very close to the control samples.

The viscosity test has enhanced the understanding of the rheological behavior of the samples. Although the addition of fiber has slightly increased the viscosity of the samples compared to the control sample without any fiber, the results are within the range for typical values. The temperature susceptible slope shows that the pattern of viscosity changed with temperature. In the case of PMB A10E, C320, and C170 it was found that when the samples are modified with 0.5% CBs, viscosity increased significantly because fibers work as reinforcement in the binder mixture. It can be concluded from these results that 0.3% CBs as fiber can be used to modify bitumen without compromising the required properties for use in the construction of flexible pavements.

An improved binder drain off property was observed when bitumen was modified with CBs. This will save the loss of bitumen during transportation from plant to site and in the mixing of asphalt concrete. During the construction of flexible pavements, 4–7% of bitumen is used as a binder based on the type and requirement of the road. A typical density of asphalt concrete is 2000–2400 kg/m^3^. This means that around 150 Tons of CBs will be recycled in a sustainable way to construct a typical 100 km strip of a two-lane roadway as the typical thickness of SMA layer is 35–45 mm. This method can contribute to solving the environmental pollution problem of cigarette butts. Cities around the world spend millions of dollars on the collection and disposal of cigarette butts. Successful implementation of this method of recycling will turn waste cigarette butts into a valuable construction material which can be utilized in the construction of roads and highways, which is the largest sector of the world in terms of asset management.

The results associated with the physical and rheological properties of CBs modified bitumen showed that CBs could be used as a fiber modifier for bitumen. Further rheological investigation including laboratory tests on different classes of bitumen and aged bitumen modified with CBs is recommended.

## Figures and Tables

**Table 1:** Typical properties of bitumen used in this research (adapted from industry approved standards) [42,43,44,45].

Sample Type	Penetration Test (dm)	Softening Point (°C)	Viscosity at 135 °C (Pa.s)
PMB A10E	60–70 (Typical)	90 (Minimum)	5 (Typical)
Bitumen C320	40 (Minimum)	45 (Minimum)	0.53 (Typical)
Bitumen C170	62 (Minimum)	40 (Minimum)	0.40 (Typical)

**Table 2:** Blending approach 1 for bitumen modified with cellulose fiber and cigarette butts.

Type of Bitumen	Type of Fiber	% of Fiber	Mass of Bitumen (g)	Mass of Fiber (g)	Mass of Final Blend (g)
A10E	No fiber	0	181	0.00	181.00
	CB	0.2	197	0.39	197.39
		0.3	182	0.55	182.55
		0.4	211	0.84	211.84
		0.5	298	1.49	299.49
	Cellulose	0.3	155	0.47	155.47
C320	No fiber	0	205	0.00	205.00
	CB	0.2	201	0.40	201.40
		0.3	236	0.71	236.71
		0.4	198	0.79	198.79
		0.5	213	1.07	214.07
	Cellulose	0.3	172	0.52	172.52
C170	No fiber	0	229	0.00	229.00
	CB	0.2	194	0.39	194.39
		0.3	182	0.55	182.55
		0.4	179	0.72	179.72
		0.5	208	1.04	209.04
	Cellulose	0.3	155	0.47	155.47

**Table 3:** Blending approach 2 for bitumen modified with cellulose fiber and cigarette butts.

Type of Bitumen	Type of Fiber	% of Fiber	Mass of Bitumen (g)	Mass of Fiber (g)	Mass of Final Blend (g)
A10E	No fiber	0	204	0.00	204.00
	CB	0.2	225	0.45	225.45
		0.3	219	0.66	219.66
		0.4	190	0.76	190.76
		0.5	195	0.98	195.98
	Cellulose	0.3	170	0.51	170.51
C320	No fiber	0	197	0.00	197.00
	CB	0.2	186	0.37	186.37
		0.3	194	0.58	194.58
		0.4	218	0.87	218.87
		0.5	190	0.95	190.95
	Cellulose	0.3	187	0.56	187.56
C170	No fiber	0	142	0.00	142.00
	CB	0.2	167	0.33	167.33
		0.3	154	0.46	154.46
		0.4	161	0.64	161.64
		0.5	151	0.76	151.76
	Cellulose	0.3	159	0.48	159.48

**Table 4:** Blending approach 3 for bitumen modified with cellulose fiber and cigarette butts.

Type of Bitumen	Type of Fiber	% of fiber	Mass of Bitumen (g)	Mass of Fiber (g)	Mass of Final Blend (g)
A10E	No fiber	0	181	0.00	204.00
	CB	0.2	197	0.45	225.45
		0.3	182	0.66	219.66
		0.4	211	0.76	190.76
		0.5	298	0.98	195.98
	Cellulose	0.3	155	0.51	170.51
C320	No fiber	0	205	0.00	197.00
	CB	0.2	201	0.37	186.37
		0.3	236	0.58	194.58
		0.4	198	0.87	218.87
		0.5	213	0.95	190.95
	Cellulose	0.3	172	0.56	187.56
C170	No fiber	0	162	0.00	162.00
	CB	0.2	149	0.30	149.30
		0.3	165	0.50	165.50
		0.4	187	0.75	187.75
		0.5	148	0.74	148.74
	Cellulose	0.3	161	0.48	161.48

**Table 5:** Penetration and softening point results.

Type of Bitumen	Type of Fiber	% of Fiber	Blending Approach 1		Blending Approach 2		Blending Approach 3		Average Result	
			Penetration Test (dmm)	Softening Point (°C)	Penetration Test (dmm)	Softening Point (°C)	Penetration Test (dmm)	Softening Point (°C)	Penetration Test (dmm)	Softening Point (°C)
A10E	No fiber	0	62.8	91.0	61.0	90.0	62.7	91.0	62.2	90.7
	CB	0.2	62.8	90.0	60.2	90.0	62.6	90.0	61.9	90.0
		0.3	62.5	89.9	60.4	89.9	62.4	89.9	61.8	89.9
		0.4	62.4	89.7	60.7	90.0	62.4	89.7	61.8	89.8
		0.5	62.0	89.3	60.9	90.2	62.0	89.3	61.6	89.6
	Cellulose	0.3	63.9	90.7	61.6	90.8	63.0	91.0	62.8	90.8
C320	No fiber	0	41.4	45.2	41.0	46.0	41.9	45.0	41.4	45.4
	CB	0.2	40.8	45.2	41.1	45.2	40.8	45.1	40.9	45.1
		0.3	42.0	45.2	41.2	45.5	42.5	45.2	41.9	45.3
		0.4	42.9	45.0	41.6	45.4	43.0	45.6	42.5	45.3
		0.5	44.2	45.2	42.0	45.5	44.0	45.8	43.4	45.5
	Cellulose	0.3	39.8	45.9	40.3	45.9	40.0	45.3	40.0	45.7
C170	No fiber	0	66.0	42.3	62.0	41.0	62.0	41.0	63.3	41.4
	CB	0.2	62.8	42.8	61.0	40.7	61.0	40.7	61.6	41.4
		0.3	61.8	42.6	61.4	40.2	61.4	40.2	61.5	41.0
		0.4	61.6	42.2	61.3	40.2	61.3	40.2	61.4	40.9
		0.5	60.2	42.0	61.2	40.1	61.2	40.1	60.8	40.7
	Cellulose	0.3	60.8	42.8	61.5	40.9	61.5	40.9	61.3	41.5

**Table 6:** Viscosity test results of the samples.

Type of Bitumen	Type of Fiber	% of Fiber	Viscosity (Pa.s)							
			Blending Approach 1		Blending Approach 2		Blending Approach 3		Average Viscosity	
			135 °C	165 °C	135 °C	165 °C	135 °C	165 °C	135 °C	165 °C
A10E	No fiber	0	6.43	1.03	6.53	1.03	6.5	1.02	6.49	1.03
	CB	0.2	6.23	1.02	6.24	1.02	6.21	1.02	6.23	1.02
		0.3	6.25	1.02	6.25	1.09	6.22	1.12	6.24	1.08
		0.4	7.01	1.26	7.05	1.29	7.02	1.25	7.03	1.27
		0.5	7.28	1.32	7.4	1.34	7.37	1.33	7.35	1.33
	Cellulose	0.3	6.59	1.01	6.91	1.02	6.88	1.05	6.79	1.03
C320	No fiber	0	0.78	0.36	0.72	0.35	0.74	0.35	0.75	0.35
	CB	0.2	0.82	0.37	0.77	0.37	0.76	0.34	0.78	0.36
		0.3	0.85	0.38	0.85	0.37	0.8	0.42	0.83	0.39
		0.4	0.91	0.38	0.91	0.41	0.91	0.51	0.91	0.43
		0.5	1.08	0.43	1.05	0.44	1.1	0.7	1.08	0.52
	Cellulose	0.3	0.77	0.36	0.76	0.36	0.74	0.04	0.76	0.25
C170	No fiber	0	0.65	0.31	0.65	0.31	0.65	0.31	0.65	0.31
	CB	0.2	0.67	0.35	0.67	0.35	0.67	0.35	0.67	0.35
		0.3	0.68	0.35	0.68	0.35	0.68	0.35	0.68	0.35
		0.4	0.73	0.39	0.73	0.39	0.73	0.39	0.73	0.39
		0.5	1.1	0.44	1.1	0.44	1.1	0.44	1.10	0.44
	Cellulose	0.3	0.66	0.34	0.66	0.34	0.66	0.34	0.66	0.34

**Table 7:** Materials used for the preparation of stone mastic asphalt (SMA) for binder drain off test.

Materials		% of Materials for SMA Mix (by Weight)	
		With Fiber	Without Fiber
Aggregates (retained in the sieve)	9.5 mm	4.36	4.36
	6.7 mm	52.32	52.32
	4.7 mm	2.62	2.62
	2.36 mm	8.72	8.72
	Fines	19.18	19.48
Filler (limestone)		6	6
Fiber		0.3	-
Bitumen		6.5	6.5

**Table 8:** Binder drain off test results.

Asphalt Type	Fiber Used	Type of Bitumen	Mass, g			Binder Drain Off (%)	Maximum Allowable Drain Off (%)
			Asphalt	Remaining Binder in the Beaker	Mineral Aggregates Present in Remaining Binder		
SMA	0% Fiber	PMB A10E	896.9	4.1	0.7	0.38	0.3%
SMA	0.3% cellulose fiber	PMB A10E	851.8	8.9	6.4	0.29	
SMA	0.3% CBs fiber	PMB A10E	939	3.1	0.7	0.26	
